# Symptoms of Avoidant/Restrictive Food Intake Disorder among 2–10-Year-Old Children: The Significance of Maternal Feeding Style and Maternal Eating Disorders

**DOI:** 10.3390/nu14214527

**Published:** 2022-10-27

**Authors:** Anna Brytek-Matera, Beata Ziółkowska, Jarosław Ocalewski

**Affiliations:** 1 Institute of Psychology, University of Wrocław, 50-527 Wrocław, Poland; 2 Faculty of Psychology, Kazimierz Wielki University, 85-064 Bydgoszcz, Poland

**Keywords:** avoidant/restrictive food intake disorder, feeding disorders, eating disorders, children, developmental disorders, feeding

## Abstract

The aim of the present study was to investigate whether the feeding style and core behavioral features of eating disorders of mothers are related to the symptoms of Avoidant/Restrictive Food Intake Disorder (ARFID) among their children. This study involved 207 mothers of children aged 2 to 10 years (*M*_age_ = 5.82 ± 2.59 years), of which 19.32% were children with neurodevelopmental disorders and 22.71% were children with chronic diseases (e.g., allergy, asthma, diabetes). The mothers were asked to complete the ARFID Parents Questionnaire–Parents Report (ARFID-Q-PR), the Parental Feeding Style Questionnaire (PFSQ) and the Eating Disorder Examination Questionnaire (EDE-Q). Our findings revealed that both the maternal feeding style and core behavioral features of eating disorders were associated with ARFID symptoms among their 2–10-year-old children. While biological factors increase the risk of feeding/nutrition difficulties, the maternal attitude towards feeding and eating behavior may play a relevant role in children’s eating behavior.

## 1. Introduction

Avoidant/Restrictive Food Intake Disorder (ARFID) is a complex eating disorder that cannot be explained by culture, religion, weight loss or other diseases/disorders [1]. ARFID manifests itself as an aversion to the selected sensory properties of food (e.g., texture, smell, taste), a fear of unpleasant consequences of eating (e.g., nausea, pain, choking) and a lack of interest in food [1]. 

As a result of refusing/restricting food intake, a child’s weight decreases or does not increase (although sometimes ARFID is diagnosed in overweight children) [2]. In addition, there is hypotrophy, vitamin, micro- and macro-element deficiencies, weakness, dizziness, fainting, abdominal pain, constipation, hypothermia, dry and pale skin, and lanugo [3,4]. In the case of children, the consequence of significant nutritional deficiencies is also growth inhibition and abnormal general development [5]. ARFID also impairs a child’s psychosocial functioning, which makes them systematically avoid social gatherings (e.g., birthdays, holidays, school trips, outings to stores or restaurants) [6,7]. Short stature, accompanied by atypical behavior during an eating situation, may lower self-esteem and, at the same time, be the cause of exclusion from the peer group [8].

Constant avoidance of social relationships negatively impacts self-esteem in children and negatively affects their mental state [9,10].

Due to the negative consequences of ARFID on the health and proper development of a child, it is necessary to look for the mechanisms of this disorder in order to counteract its development and its multiple, sometimes serious, consequences.

### 1.1. Complex Etiology of ARFID in Children

The etiology and mechanism of ARFID has not been fully understood, but it is certainly a polyetiological disorder. According to Ellis et al. [11], parental pressure to eat, children’s greater vulnerability to disgust and aversive eating experiences can result in picky eating (PE) behavior, which in turn could be associated with ARFID. ARFID is generally associated with eating disorders (Great Ormond Street Criteria, i.e., selective eating, emotional eating avoidance disorders and functional dysphagia) [12]. Therefore, for example, children born prematurely, suffering from genetic, nervous and digestive system diseases, more often suffer from nutritional problems [13,14]. Eating disorders, including ARFID, appear more often in children with neurodevelopmental disorders and intellectual disabilities than in the general population [15]. Moreover, these disorders can be caused by low muscle tone affecting oral motor skills [16]. It has also been proven that feeding and eating disorders in children are related to their difficult temperament and an increased level of physiological arousal [17,18,19]. A recent study by Katzman et al. [20] found that sex and age are important in the manifestation of ARFID symptoms: boys more often than girls refuse to eat because of the sensory properties of food, and younger children (5–10 years old) more often than older ones have sensory reluctance and a lack of interest in eating. There is also evidence of genetic determinants of appetite regulation and taste preferences in early life [21]. Although the results of twin studies confirm that eating disorders (mainly both the restricting and purging subtypes of anorexia nervosa) are of genetic origin [22], the environment in which a child is raised plays the greatest role in expressing the basic genetic predisposition.

A higher occurrence of neurodevelopmental disorders has been found In patients with ARFID compared to general population estimates [23]. The prevalence of autism spectrum disorder (ASD) has been estimated at 3–23% among children and adolescents with ARFID [24,25]. ARFID–ASD comorbidity might be explained by the fact that ARFID and ASD comprise common symptoms such as sensory sensitivity, repetitive behaviors, rigidity and high arousal. These symptoms, individually or together, might predispose a child to developing ARFID [26].

### 1.2. Psychosocial Factors and Maternal Feeding Behaviors

Parents play a key role in modeling nutritional behavior [27]. In turn, meals are extremely important in children’s lives not only in terms of nutrition. The experiences related to feeding and eating influence their sense of security, inner sense of self, autonomy and initiative [28]. Usually, a baby’s attitude to food largely depends on the mother’s mental and physical condition. It turns out that children of mothers with depression reveal difficulties in feeding/eating more often than children of healthy mothers [29,30], due to the negative affect and non-reactivity of the mother [18,31]. The results of previous studies have also demonstrated that problems with feeding infants can coexist with the phobic anxiety, depressive disorders, somatization, paranoid thoughts and mental stress of their mothers [32,33,34,35].

The factors that influence children’s feeding are, i.e., maternal eating style and maternal eating disorders. A Norwegian study demonstrated [36] that mothers diagnosed with bulimia nervosa and compulsive eating more often reported eating problems of their children than mothers without eating disorders. These findings may result from the fact that, due to their own experiences, mothers are concerned about whether they properly nourish their child and whether the child will have an eating disorder [37]. Moreover, mothers with eating disorders do not want their children, especially girls over 2 years of age, to overeat [37] or even try to help them lose weight [38].

It has been found that mothers who had experienced eating disorders themselves preferred a controlling style at mealtimes and their children had difficulty in eating [39,40]. Similar results were obtained by Birch and Davison [41]: parents who had problems with eating control preferred a controlling feeding style for their child to prevent the development of overweight. In turn, Braden et al. [42] demonstrated that mothers with binge eating tend to offer food to their child to soothe their negative emotions. The previous longitudinal studies of both mothers and fathers with binge eating have shown [43] that they had maladaptive relationships with their babies during feeding, as a result of which their babies developed emotional and behavioral problems.

To sum up, problems in the psychosocial functioning of a mother influence her interaction with the baby during feeding, which is expressed, inter alia, in the style of feeding. However, it should not be forgotten that the quality of the mother–child relationship, also during feeding, is determined by the features of both participants of the dyad [44].

### 1.3. Study Objective

The aim of the present study was to determine whether maternal feeding style and core behavioral features of eating disorders among mothers are related to the symptoms of ARFID among their children, aged 2 to 10 years.

To be able to address the scientific gap on this topic and its relevance in public health, new research in this area providing useful insights on eating pathology (especially in the context of ARFID) is necessary. Therefore, we supposed that maternal feeding style and symptoms of eating disorders may be related to a preference for a specific feeding style of a child, which may in turn play a role in the development of ARFID symptoms in the child.

## 2. Methods

### 2.1. Participants and Procedure

The current study is part of a research project focusing on the psychological correlates of ARFID among children. The procedure of the study was described in detail in the previous work [45]. That work focused on increasing the availability of validated ARFID symptom measures for children, in Polish, by developing a new measure, the Avoidant/Restrictive Food Intake Disorder Questionnaire—Parents Report (ARFID-Q-PR), and by evaluating its measurement properties [45]. The present study, however, has concentrated on maternal feeding style and symptoms of eating disorders, and their relationship with ARFID symptoms in children.

The research project was carried out between 2020 and 2021, partly online, due to the SARS-CoV-2 pandemic. The research was initially conducted in person, with recruitment in institutions such as nurseries, kindergartens, children’s clubs and schools in different regions in Poland (Greater Poland, Silesia, Lower Silesia and the Kuyavian-Pomeranian Voivodeship). Then, due to the epidemiological situation, an electronic version of the study was prepared using Google Forms, and a link to it was made available on online forums for parents from the same regions. In both recruitment procedures, caregivers were assured of the voluntary nature of participation and confidentiality of the research, and provided informed consent.

Some parents had more than one child, but caregivers reported only one child aged 2 to 10 for the study.

The research project was approved by the Scientific Research Ethics Committee, located at the Faculty of Psychology of Kazimierz Wielki University in Bydgoszcz, Poland (consent no. 2/5.11.2019).

The study sample consisted of 207 mothers of 2–10-year-old children. The sample consisted of mothers who participated in the ARFID-Q-PR validation study (*n* = 167) [45] and additional data were collected in this study from mothers of children with developmental disorders (e.g., autism spectrum disorders, sensory disorders) (*n* = 40). Among all mothers, 44.92% (*n* = 93) had a daughter and 55.07% (*n* = 114) had a son. (Our project focused only on mothers. We used conscious selection to make sure the sample was consistent with who was feeding the baby). The mean age of the children was 5.82 years (SD = 2.59) (Table 1).

Among all children, 19.32% consisted of children with developmental disorders (e.g., autism spectrum disorders (*n* = 17), sensory disorders) and 22.71% consisted of children with chronic diseases (e.g., allergy, asthma, diabetes).

### 2.2. Measures

#### 2.2.1. Demographic and Medical Characteristics

The mothers of the children were asked to answer questions concerning their child’s date of birth, sex, height and weight, chronic somatic diseases, neurodevelopmental, and mental disorders and intellectual disability.

#### 2.2.2. The ARFID Questionnaire—Parents Report

The ARFID Questionnaire—Parents Report (ARFID-Q-PR) was developed in Poland [45]. The questionnaire consists of 14 items (e.g., “My child does not eat some dishes because he or she thinks they have an unpleasant consistency/texture”, “My child spits out food”). The parents reported on their child’s behavior. They answered each item on a five-point scale (“almost never”–“rarely”–“occasionally”–“often”–“almost always”). The three-factor structure of the ARFID-Q-PR was confirmed as: Attitude to food (AF), Justification for restriction (JR) and Somatic condition (SC). The first factor contains a group of statements describing the child’s behavior towards food; the second concerns the child’s motives for rejecting food or the conditions under which the child consumes it; and the third concerns the somatic effects of dietary restrictions. The Cronbach’s alpha value across the whole questionnaire was 0.87 (Attitude to food—0.78, Justification for restriction—0.76, Somatic condition—0.75). ARFID diagnosis can occur when two conditions are met: (1) the sum of the score for the subscales, “Attitude to food” and “Justification for restriction” is at least 25 points; and (2) the sum of the subscales, “Attitude to food”, “Justification for restriction” and “Somatic condition” is at least 35 points.

#### 2.2.3. The Parental Feeding Style Questionnaire

The Parental Feeding Style Questionnaire (PFSQ) [46] consists of 27 items (e.g., “I reward my child with something to eat when he or she is well-behaved”, “I give my child something to eat to make him or her feel better when they are upset”). Parents described their feeding behavior on a five-point scale: “never”–“rarely”–“sometimes”–“often”–“always”. We used the Polish adaptation of the PFSQ [47]. The PFSQ consists of four subscales: Instrumental feeding (IF) (i.e., using food as a reward), Control (CT), Emotional feeding (EM) and Encouragement (EN). Scale scores were obtained by calculating the means of the items comprising each scale. The higher the score, the more specific the parental feeding style (i.e., IF, CT, EM EN) was. The reliability of the Polish adaptation of the PFSQ is effective and ranges from 0.69 to 0.86 [47]. These values are similar to those obtained by other researchers (e.g., [48]). In the present study, the Cronbach’s alpha value across the whole questionnaire was 0.79 (IF—0.76, CT—0.53, EM—0.81, EN—0.78).

#### 2.2.4. The Eating Disorder Examination Questionnaire

We used a seven-item version of the Eating Disorder Examination Questionnaire (EDE-Q) [49] (e.g., “Have you attempted to avoid eating any foods that you like in order to influence your shape or weight?”, “How dissatisfied have you felt about your shape?”). This self-reported tool is used to test body and food attitudes over the past 28 days. The mothers reported on their own behavior. In our study, exploratory factor analysis (EFA) confirmed the three-factor structure of the EDE-Q as: Dietary restraint (DR) (3 items), Shape/weight overvaluation (SWO) (2 items) and Body dissatisfaction (BD) (2 items). The results of the EFA analysis are presented in Table 2.

The Cronbach ‘s alpha value across the whole questionnaire was 0.88.

### 2.3. Statistical Analysis

The data were analyzed using Statistica 13 software (StatSoftPolska, Cracow, Poland). Cronbach’s alpha was used to assess the internal consistency of the ARFID Questionnaire—Parents Report (ARFID-Q-PR), the Parental Feeding Style Questionnaire (PFSQ) and the Eating Disorder Examination Questionnaire (EDE-Q). Exploratory factor analysis (EFA) was first used to detect the EDE-Q factor structure and the maximum likelihood method was adopted (the number of factors was based on the Kaiser criterion) [50]. Then, we used the standardized varimax rotation method to simplify the expression of a particular subspace in terms of just a few major items each. The difference between the groups was verified with the nonparametric Mann–Whitney U test (the PFSQ, the EDE-Q, developmental disorders and the ARFID-Q-PR), and then, Spearman’s rank correlation coefficient was used. Missing data (from the ARFID-Q-PR or measures of validity) were excluded (*n* = 9). The significance level p was set at 0.05. We also conducted a series of multiple linear variation regressions to explain the ARFID.

## 3. Results

Table 3 presents the results of Spearman’s Rho correlation test between the PFSQ, EDE-Q, ARFID-Q-PR subscales and other variables.

There were 27 children who met the ARFID criteria on the basis of the ARFID-Q-PR, which was 13.04% of the sample.

The results demonstrated that there are several statistically significant associations between the subscales measuring maternal feeding styles, maternal core behavioral features of eating disorders and ARFID symptoms among their children, as well as age, sex, somatic diseases and developmental disorders in their children. We found a weak relationship between “Dietary restraint” (EDE-Q), and “Instrumental feeding” and “Control” (PFSQ). On the other hand, “Shape/weight overvaluation” (EDE-Q) showed a moderate association with “Instrumental feeding” (PFSQ).

“Attitude to food” (ARFID-Q-PR) correlated weakly with “Control” (PFSQ), while “Justification for restriction” (ARFID-Q-PR) was moderately related to “Shape/weight overvaluation” (EDE-Q).

There were moderate correlations between the sex of the child, and “Encouragement” (PFSQ) and “Attitude to food” (ARFID-Q-PR) (i.e., a negative one). Developmental disorders correlated moderately with all dimensions of the ARFID-Q-PR and the overall score of this questionnaire.

In the first step, the PFSQ, EDE-Q and ARFID-Q-PR in the group of mothers of children with ARFID without developmental disorders were compared with the group of mothers of children with ARFID with these disorders. Among all the factors of the PFSQ, only the intensity of “Instrumental feeding” in the group of mothers of children with ARFID without developmental disorders (M = 6.90, SD = 2.53) was statistically significantly higher (U = 2668.50, *p* = 0.049) than in the group of mothers of children with ARFID with developmental disorders (M = 6.33, SD = 3.14). “Attitude to food”, “Justification for restriction” and “Somatic condition” (subscales of the ARFID-Q-PR) were statistically significantly higher in the group of mothers of children with ARFID with developmental disorders than in the group of mothers of children with ARFID without developmental disorders (for the subscale, “Attitude to food”: M = 13.13, SD = 4.00 vs. M = 9.75, SD = 3.63, U = 1733.00, *p* < 0.001, respectively; for the subscale, “Justification for restriction”: M = 10.80, SD = 4.28 vs. M = 8.51, SD = 3.11, U = 2253.00, *p* = 0.001, respectively; and for subscale, “Somatic condition”: M = 11.90, SD = 4.25 vs. M = 5.56, SD = 2.81, U = 732.50, *p* < 0.001, respectively). The severity of the total ARFID-Q-PR was also statistically significantly higher in the group of mothers of children with ARFID with developmental disorders than in the group of mothers of children with ARFID without developmental disorders (M = 35.83, SD = 11.15 vs. M = 23.83, SD = 5.72, U = 1214.50, *p* < 0.001, respectively). The statistical parameters of the comparison using the Mann–Whitney U test are presented in Table 4.

To investigate the relationship between the PFSQ, EDE-Q and ARFID-Q-PR, we conducted a series of multiple linear regressions to maximize the percentages of the result’s variance (rejection of outliers) and stepwise regression to check the change in the R^2^ of the individual model factors. We checked which factors of the PFSQ and the occurrence of developmental disorders statistically significantly explained the severity of the ARFID symptoms. As shown in Table 5, the multivariate regression model for the “Attitude to food” revealed the following statistically significant independent variables: “Instrumental feeding” (β* = −0.16, *p* = 0.045), “Control” (β* = 0.22, *p* < 0.001), “Emotional feeding” (β* = 0.18, *p* = 0.024) and “Developmental disorders” (β* = 0.37, *p* < 0.001). “Developmental disorders” explained 12% of the variability of the “Attitude to food”, while PFSQ factors, 8% (F(5199) = 10.22, *p* < 0.001). Regression for the “Justification for restriction” proved statistically significant for “Control” and “Emotional feeding” (in total, they explained 6% of the variability of the “Justification for restriction”). For the ARFID symptoms, it revealed the following statistically significant independent variables: “Instrumental feeding” (β* = −0.15, *p* = 0.041), “Control” (β* = 0.24, *p* < 0.001), “Emotional feeding” (β* = 0.15, *p* = 0.045) and “Developmental disorders” (β* = 0.52, *p* < 0.001). It is worth pointing out that “Developmental disorders” explained 25% of the variability of the ARFID symptoms, while PFSQ factors explained 9% (F(5195) = 19.72, *p* < 0.001).

In the next step, we performed a series of multiple regressions in which the independent variables were the EDE-Q factors (“Dietary restraint”, “Shape/weight overvaluation”, “Body dissatisfaction”), and “Developmental disorders”, while the dependent variables were the ARFID-Q-PR subscales (“Attitude to food”, “Justification for restriction”, “Somatic condition” and the ARFID-Q-PR total) (Table 6).

The multivariate regression model for the “Attitude to food” revealed the following statistically significant independent variables: “Dietary restraint” (β* = 0.14, *p* = 0.041), “Shape/weight overvaluation” (β* = 0.20, *p* = 0.023), “Body dissatisfaction” (β* = −0.19, *p* = 0.028) and “Developmental disorders” (β* = 0.49, *p* < 0.001). “Developmental disorders” explained 21% of the variability of the “Attitude to food”, while EDE-Q factors explained 5% (F(4194) = 17.02, *p* < 0.001). Another regression model for “Justification for restriction” had the following independent variables: “Shape/weight overvaluation”, “Body dissatisfaction” (β* = −0.20, *p* = 0.030) and “Developmental disorders” (β* = 0.37, *p* < 0.001). “Developmental disorders” explained 11% of the variability of the “Justification for restriction”, while EDE-Q factors explained 7% (F(4194) = 10.49, *p* < 0.001). In the “Somatic condition” regression model, only the variable, “Developmental disorders” was statistically significant (β* = 0.62, *p* < 0.001). This model explained 40% of the variability of the “Somatic” condition. Interestingly, for the ARFID-Q-PR total, “Shape/weight overvaluation” and “Developmental disorders” were statistically significant variables (β* = 0.17, *p* = 0.036; β* = 0.60, *p* < 0.001). “Developmental disorders” explained 34% of the variability of the ARFID-Q-PR total score and “Shape/weight overvaluation” explained 2% (F(4199) = 28.17, *p* < 0.001).

Due to the fact that some independent variables in the regression model were correlated (for instance PFSQ “Control” and PFSQ “Encouragement” with *r* = 0.40 *** or EDE-Q “Dietary restraint” and EDE-Q “Shape/weight overvaluation” with *r* = 0.51 ***), we performed a collinearity test of the variance inflation factor (VIF). All these models did not exceed a VIF > 10, which can be interpreted as there being poor relationships between the independent variables (additionally, the VIF for individual predictors did not exceed the value of 2) [51].

## 4. Discussion

The objective of the present study was to investigate the relationship between maternal feeding style and the core behavioral features of eating disorders, and the symptoms of ARFID among their 2–10-year-old children.

ARFID is an eating disorder whose determinants and mechanism have not been well understood so far. Both the biological and psychosocial determinants of this disorder are indicated. There is evidence that ARFID, like other feeding and nutritional difficulties, is the most common in the clinical population—i.e., children with developmental disorders (see [52,53,54]). Our results confirmed these findings. Using maternal perspectives, children diagnosed with autism spectrum disorders (*n* = 17) obtained significantly higher results in the ARFID-Q-PR than healthy children, including in “Attitude to food”, “Justification for restriction”, “Somatic state” and the global score of the ARFID-Q-PR. Previous studies have revealed that somatic diseases are associated with ARFID, and appear more often in children with gastric and nervous system diseases than in healthy children (cf., [55,56,57]). We found no correlation between the symptoms of ARFID and the somatic health of the children with ARFID. However, the qualitative analysis of the obtained results indicated that according to mothers’ descriptions, their children with ARFID suffered from allergies, asthma and diabetes. Perhaps these disease entities are not directly related to the development of ARFID in children, but this assumption still needs to be verified.

Our findings demonstrated that mothers of children without developmental disorders used instrumental nutrition style slightly more often than mothers of children with developmental disorders. Therefore, it can be supposed that the presence or absence of developmental disorders is not significantly related to the way in which mothers feed their children. We did not find any research comparing the styles of children’s nutrition due to the occurrence of developmental disorders. However, researchers [58] indicate the need for proper nutrition in children with autism, e.g., for weight control. Moreover, they emphasize that children with autism have problems with, e.g., chewing; therefore, there are significant differences in their nutritional status compared to children without developmental disorders [59].

We found that the mothers of children without developmental disorders more often used food as a reinforcement in raising their children. Perhaps this is because for children with feeding difficulties, including ARFID, food is not of a satisfactory value. Moreover, our results showed that “Attitude to food” (ARFID-Q-PR) had a positive relationship with “Control” (PFSQ), which means that the more controlling the maternal feeding style, the greater the severity of ARFID symptoms in their children. In addition, we found that less “Symptoms of food refusal” appeared in children whose mothers encouraged them to try to eat (PFSQ). Moreover, factors related to children’s developmental disorders and their eating style explained the severity of ARFID, but it was to a much greater extent affected by developmental disorders.

To the best of our knowledge, there is a lack of research on the relationship of ARFID symptoms in children and maternal feeding style. Nevertheless, the previous study demonstrated that family meals are described as extremely frustrating and stressful for children with ARFID, and reinforce their behaviors [59,60]. Another study revealed that babies with feeding problems are restless and hyperactive at mealtimes [8]. As such, caregivers often try different strategies to overcome their child’s feeding or eating problems, but the way they offer food can escalate problems [61,62]. Perhaps the pathway of ARFID development in children with developmental disorders results primarily from biological conditions (e.g., sensory sensitivity, temperament), and secondarily from social factors (i.e., the way the caregiver acts while feeding the children), which may intensify the ARFID symptoms.

There are limited data on the relationship between the core behavioral features of eating disorders among mothers of children and ARFID symptoms in their children. In our study, we found that there is a statistically significant moderately positive relationship between “Justification for restriction” (ARFID-Q-PR) and “Shape/weight overvaluation” (EDE-Q). In other words, the reasons for the children’s reluctance to eating are associated with self-satisfaction with weight and body shape among their mothers. We also found out that mothers who scored higher on “Dietary restraint” (EDE-Q) more often used food to reinforce their child (“Instrumental feeding” (PFSQ)) and exerted more control while eating (“Control” (PSFQ)). Moreover, mothers whose self-satisfaction was based on “Shape/weight overvaluation” (EDE-Q) often used food as a reinforcement in their children’s socialization process (“Instrumental feeding” (PFSQ)). Multiple regression analyses showed that “Attitude to food” (ARFID-Q) was primarily explained by developmental disorders in children (21% variability), but also by EDE-Q factors (5% variability). Similarly, the “Justification for restriction” (ARFID-Q) was explained both by child developmental disorders (11% variability) and simultaneously by EDE-Q factors (7% variability). The overall sum of the ARFID-Q-PR was explained by developmental disorders in 34% and by “Shape/weight overvaluation” (EDE-Q) in 2%.

Our results are consistent with previous studies. A prospective study [39] showed that mothers with eating disorders fed their children less regularly than healthy mothers, used food for non-nutritional purposes, and were concerned about their daughters’ weight from the age of 2 years. The 5-year-old children, on the other hand, showed significantly more problems with eating than the children of healthy mothers. Blissett and Haycraft [63] indicated that the symptoms of eating disorders in parents are associated with restricting eating in their children. Meanwhile, excessive childhood eating control by caregivers may contribute to the transmission of eating disorders to children (cf., also [64]). Hoffman et al. showed [65] that mothers with eating disorders more often than healthy mothers used restrictions in feeding their children, and also limited processed foods in their children’s diets and preferred organic ones.

The present study is not without its limitations. First, due to the cross-sectional design, we were unable to draw causal inferences. The second limitation is the homogeneity of the sample (only mothers of children); therefore, future studies should include the fathers of children. The third is a lack of information about the mental conditions and disorders of the mothers of the children, and their influence on maternal feeding style and eating pathology. The fourth limitation is that the study was conducted partly online and partly through real contact, so the study conditions were not the same for all mothers. Fifthly, participation in the project during the COVID-19 pandemic was also not convenient for the participants.

## 5. Conclusions

Our results demonstrate that maternal feeding style and the core behavioral features of eating disorders were related to the symptoms of ARFID among their 2–10-year-old children.

Neurodevelopmental disorders (i.e., diagnosis of autism spectrum disorders) are undoubtedly a risk factor for the development of feeding/eating difficulties, including ARFID. It is interesting to notice, however, what psychosocial reasons may affect the development of this disorder, especially in children with ARFID without developmental disorders. If a mother is the main feeder and her relationship with food and her body is disturbed, she may model her child’s inappropriate relationship with food. Research in this direction may help to elucidate persistent feeding problems in children despite receiving adequate food therapy, in children with developmental disorders, and in the treatment of comorbid conditions.

## Figures and Tables

**Table 1:** Characteristics of children (*n* = 207).

	Children without Developmental Disorders (*n* = 167)	Children with Developmental Disorders (*n* = 40)	All Children (*n* = 207)
Age M (SD) in years	5.77 (2.47)	5.99 (2.69)	5.82 (2.59)
Boys (*n* = 114) M (SD)	5.31 (2.33)	6.20 (2.71)	5.54 (2.45)
Girls (*n* = 93) M (SD)	6.25 (2.53)	5.43 (2.67)	6.16 (2.55)
Chronic illness other than developmental disorders *n* (*%*)	31 (18.56%)	16 (40.00%)	47 (22.71%)

Note: *n* = sample size; M = mean value; SD = standard deviation value.

**Table 2:** EFA factor analysis of the Eating Disorder Examination Questionnaire (*n* = 207).

Item	EFA Factor Loading		
	Shape/Weight Overvaluation (Cronbach’s Alpha = 0.97)	Dietary Restraint (Cronbach’s Alpha = 0.89)	Body Dissatisfaction (Cronbach’s Alpha = 0.90)
1. Restraint over eating	0.064	0.861	0.204
2. Food avoidance	0.242	0.874	0.123
3. Dietary rules	0.191	0.901	0.101
4. Importance of weight	0.898	0.216	0.335
5. Importance of shape	0.886	0.207	0.367
6. Dissatisfaction with weight	0.323	0.126	0.891
7. Dissatisfaction with shape	0.332	0.229	0.857
Out condition	1.842	2.476	1.904
Participation	0.263	0.354	0.272

Note: EFA *=* exploratory factor analysis; numbers represent raw factor loadings; *n =* sample size.

**Table 3:** Pearson’s R correlations: ARFID-Q-PR, EDE-Q, PFSQ, age, sex, chronic diseases, means and standard deviations of individual variables.

	1	2	3	4	5	6	7	8	9	10	11	12	13	14
1. PFSQ Instrumental feeding	-													
2. PFSQ Control	0.03	-												
3. PFSQ Emotional feeding	0.56 ***	0.01	-											
4. PFSQ Encouragement	0.10	0.40 ***	0.11	-										
5. EDE-Q Dietary restraint	0.14 *	0.17 *	0.12	0.11	-									
6. EDE-Q Shape/weight overvaluation	0.18 **	0.11	0.14 *	0.10	0.51 ***	-								
7. EDE-Q Body dissatisfaction	0.16 *	0.17 *	0.10	0.07	0.48 ***	0.67 ***	-							
8. ARFID-Q-PR Attitude to food	−0.06	0.14 *	0.08	0.06	0.05	0.11	0.03	-						
9. ARFID-Q-PR Justification for restriction	−0.01	0.01	0.07	0.04	0.05	0.19 **	0.06	0.65 ***	-					
10. ARFID-Q-PR Somatic condition	−0.08	0.03	−0.05	0.04	−0.04	−0.08	0.07	0.14 *	0.11	-				
11. ARFID-Q-PR Total	−0.07	0.10	0.04	0.07	0.02	0.09	0.07	0.84 ***	0.79 ***	0.50 ***	-			
12. Age	−0.02	−0.15 *	−0.14 *	−0.06	0.08	0.05	0.02	−0.08	0.15 *	<0.01	<0.01	-		
13. Sex	0.13	−0.02	−0.08	−0.05	0.18 **	0.07	<0.01	−0.21 **	−0.01	0.03	−0.11	0.13	-	
14. Chronic diseases	0.01	0.04	−0.06	−0.05	0.07	<0.01	0.05	0.07	0.13	0.13	0.10	0.25 ***	0.05	-
15. Developmental disorders	−0.14 *	−0.04	−0.06	−0.02	−0.11	−0.08	<0.01	0.33 ***	0.22 ***	0.54 ***	0.44 ***	0.03	−0.17 *	0.20 **
M	6.79	33.89	9.00	30.77	8.01	7.37	7.19	10.41	8.96	6.79	26.15	5.82	-	-
SD	2.66	3.94	3.29	4.71	6.20	4.35	4.11	3.94	3.48	4.01	8.51	2.51	-	-

Note: * *p* < *0.05*, ** p < *0.01*, *** p < *0.001*.

**Table 4:** Results of the Mann–Whitney U test as a comparison between the PFSQ, EDE-Q and ARFID-Q-PR in two groups: mothers of children without developmental disorders and mothers of children with developmental disorders.

Variables	Mothers of Children without Developmental Disorders (*n* = 167)		Mothers of Children with Developmental Disorders (*n* = 40)		Mann–Whitney U Test	*Z* Value	*p*
	*M*	*SD*	*M*	*SD*			
1. PFSQ Instrumental feeding	6.90	2.53	6.33	3.14	2668.50	1.97	0.049
2. PFSQ Control	34.04	3.29	33.28	5.96	3127.50	0.62	0.533
3. PFSQ Emotional feeding	9.08	3.29	8.65	3.29	3038.50	0.88	0.376
4. PFSQ Encouragement	30.94	4.33	30.08	6.45	3263.00	0.22	0.822
5. EDE-Q Dietary restraint	8.32	6.34	6.70	5.50	2817.50	1.53	0.125
6. EDE-Q Shape/weight overvaluation	7.55	4.40	6.63	4.08	2945.00	1.16	0.246
7. EDE-Q Body dissatisfaction	7.20	4.16	7.13	3.92	3327.50	0.04	0.972
8. ARFID-Q-PR Attitude to food	9.75	3.63	13.13	4.00	1733.00	−4.72	<0.001
9. ARFID-Q-PR Justification for restriction	8.51	3.11	10.80	4.28	2253.00	−3.19	0.001
10. ARFID-Q-PR Somatic condition	5.56	2.81	11.90	4.25	732.50	−7.66	<0.001
11. ARFID-Q-PR Total	23.83	5.72	35.83	11.15	1214.50	−6.24	<0.001

Note: Mann–Whitney U test—the result of the Mann–Whitney U test; *n* = sample size; *M* = mean value; *SD* = standard deviation value.

**Table 5:** The results of the multivariate regression analysis for the ARFID-Q-PR.

Variables	Attitude to Food				Justification for Restriction				Somatic Condition				ARFID-Q-PR Total Score			
	*R*^2^ = 0.20; *R*^2^ *Adjusted* = 0.18 *F*(5199) = 10.22; *p <* 0.001 *VIF* = 7.11				*R*^2^ = 0.13; *R*^2^ *Adjusted* = 0.11 *F*(5191) = 5.76; *p <* 0.001 *VIF* = 6.97				*R*^2^ = 0.40; *R*^2^ *Adjusted* = 0.39 *F*(5201) = 27.24; *p <* 0.001 *VIF* = 7.12				*R*^2^ = 0.34; *R*^2^ *Adjusted* = 0.33 *F*(5195) = 19.72; *p* < 0.001 *VIF* = 6.98			
	β*	*t*	*p*	*R* ^2^	β*	*t*	*p*	*R* ^2^	β*	*t*	*p*	*R* ^2^	β*	*t*	*p*	*R* ^2^
Absolute term		0.857	0.392			0.92	0.361			1.23	0.218			1.70	0.091	
Instrumental feeding	−0.16	−2.01	0.045	0.02	−0.03	−0.38	0.704	<0.01	−0.05	−0.77	0.441	<0.01	−0.15	−20.05	0.041	0.01
Control	0.22	20.99	0.003	0.05	0.18	2.20	0.029	0.04	0.08	10.28	0.201	<0.01	0.24	3.50	<0.001	0.07
Emotional feeding	0.18	2.27	0.024	0.01	0.17	1.99	0.047	0.02	−0.01	−0.10	0.917	<0.01	0.15	2.02	0.045	0.01
Encouragement	−0.01	−0.04	0.97	<0.01	0.02	0.24	0.812	<0.01	0.03	0.49	0.62	<0.01	0.03	0.38	0.701	<0.01
Developmental disorders	0.37	5.84	<0.001	0.12	0.29	4.21	<0.001	0.07	0.63	11.47	<0.001	0.39	0.52	8.74	<0.001	0.25

Note: *R*^2^—the coefficient of determination (*R*^2^ for individual factors was given by stepwise regression analysis); *Adjusted R*^2^—the corrected coefficient of determination; β*—a standardized regression coefficient; *t* —the independent samples t-Test

**Table 6:** The results of the multivariate regression analysis for the ARFID-Q-PR.

Variables	Attitude to Food				Justification for Restriction				Somatic Condition				ARFID-Q-PR Total Score			
	*R*^2^ = 0.26; *R*^2^ *Adjusted* = 0.24 *F*(4194) = 17.02; *p* < 0.001 *VIF* = 6.11				*R*^2^ = 0.18; *R*^2^ *Adjusted* = 0.16 *F*(4194) = 10.69; *p* < 0.001 *VIF* = 6.14				*R*^2^ = 0.40; *R*^2^ *Adjusted* = 0.39 *F*(4202) = 33.76; *p* < 0.001 *VIF* = 6.17				*R*^2^ = 0.36; *R*^2^ *Adjusted* = 0.35 *F*(4199) = 28.17; *p* < 0.001 *VIF* = 6.20			
	β*	*t*	*p*	*R* ^2^	β*	*t*	*p*	*R* ^2^	β*	*t*	*p*	*R* ^2^	β*	*t*	*p*	*R* ^2^
Absolute term		17.37	<0.001			14.57	<0.001			10.83	<0.001			19.90	<0.001	
Dietary restraint	0.14	2.06	0.041	0.02	0.08	1.04	0.298	<0.01	−0.02	−0.38	0.704	<0.01	0.06	0.99	0.320	<0.01
Shape/weight overvaluation	0.20	2.29	0.023	0.01	0.32	3.53	<0.001	0.05	−0.09	−1.23	0.219	<0.01	0.17	2.11	0.036	0.02
Body dissatisfaction	−0.19	−2.22	0.028	0.02	−0.20	−2.18	0.030	0.02	0.14	1.81	0.072	<0.01	−0.07	−0.88	0.376	<0.01
Developmental disorders	0.49	7.87	<0.001	0.21	0.37	5.56	<0.001	0.11	0.62	11.20	<0.001	0.39	0.60	10.49	<0.001	0.34

Note: *R*^2^—the coefficient of determination; *Adjusted R*^2^—the corrected coefficient of determination; β*—a standardized regression coefficient; *t* —the independent samples t-Test; *VIF* = variance inflation factor.

## Data Availability

The dataset used during the current study is available from the corresponding author upon reasonable request.

## References

[1] American Psychiatric Association (2013). Diagnostic and Statistical Manual of Mental Disorders.

[2] Kerem L., Van De Water A., Kuhnle M., Harshman S., Hauser K., Eddy K., Becker K., Misra M., Micali N., Thomas J. (2021). Neurobiology of Avoidant/Restrictive Food Intake Disorder in Youth with Overweight/Obesity Versus Healthy Weight. J. Clin. Child Adolesc. Psychol..

[3] Białek-Dratwa A., Michalak A., Grot A., Krupa-Kotara K. (2022). Complementary feeding with traditional and baby led weaning (BLW) methods—Assessment of selected aspects of infant’s diet. J. Educ. Health Sport.

[4] Mammel K.A., Ornstein R.M. (2017). Avoidant/restrictive food intake disorder: A new eating disorder diagnosis in the diagnostic and statistical manual 5. Curr. Opin. Pediatr..

[5] Grubb L.K. (2021). Avoidant Restrictive Food Intake Disorder-What Are We Missing? What Are We Waiting for?. JAMA Pediatr..

[6] Claudino A.M., Pike K.M., Hay P., Keeley J.W., Evans S.C., Rebello T.J., Bryant-Waugh R., Dai Y., Zhao M., Matsumoto C. (2019). The classification of feeding and eating disorders in the ICD-11: Results of a field study comparing proposed ICD-11 guidelines with existing ICD-10 guidelines. BMC Med..

[7] Katzman D., Stevens K. (2014). Redefining feeding and eating disorders: What is avoidant/restrictive food intake disorder?. Paediatr. Child Health.

[8] Dovey T.M., Kumari V., Blissett J. (2019). Eating behaviour, behavioural problems and sensory pro fi les of children with avoidant / restrictive food intake disorder (ARFID), autistic spectrum disorders or picky eating: Same or different?. Eur. Psychiatry.

[9] Davis C., Ng K.C., Oh J.Y., Baeg A., Rajasegaran K., Chew C.S. (2020). Caring for Children and Adolescents With Eating Disorders in the Current Coronavirus 19 Pandemic: A Singapore Perspective. J. Adolesc. Health.

[10] Nicely T.A., Lane-Loney S., Masciulli E. (2014). Prevalence and characteristics of avoidant/ restrictive food intake disorder in a cohort of young patients in day treatment for eating disorders. J. Eat. Disord..

[11] Ellis J.M., Galloway A.T., Webb R.M. (2016). Recollections of pressure to eat during childhood, but not picky eating, predict young adult eating behavior. Appetite.

[12] Nicholls D., Chater R., Lask B. (2000). Children into DSM don’t go: A comparison of classification systems for eating disorders in childhood and early adolescence. Int. J. Eat. Disord..

[13] Chatoor I., Hommel S., Sechi C., Lucrelli L. (2018). Development of the parent—Child play scale for use in children with feeding disorders. Infant Ment. Health J..

[14] Bąbik K., Rybak A. (2017). Trudności w Karmieniu u Dzieci. (w:) A. Horvath, Szajewska (red.) Żywienie i Leczenie Żywieniowe Dzieci i Młodzieży.

[15] Coglan L., Otasowie J. (2019). Avoidant/restrictive food intake disorder: What do we know so far?. BJ Psych. Adv..

[16] Bryant-Waugh R., Markham L., Kreipe R., Walsh T. (2010). Feeding and Eating Disorders in Childhood. Int. J. Eat. Disord..

[17] Aviram I., Atzaba-Poria N., Pike A., Meiri G., Yerushalmi B. (2015). Mealtime Dynamics in Child Feeding Disorder: The Role of Child Temperament, Parental Sense of Competence, and Paternal Involvement. J. Pediatr. Psychol..

[18] Lucarelli L., Ammaniti M., Porreca A., Simonelli A. (2017). Infantile Anorexia and Co-parenting: A Pilot Study on Mother–Father–Child Triadic Interactions during Feeding and Play. Front. Psychol..

[19] Råstam M., Täljemark J., Tajnia A., Lundström S., Gustafsson P., Lichtenstein P., Gillberg C., Anckarsäter H., Kerekes N. (2013). Eating problems and overlap with ADHD and autism spectrum disorders in a nationwide twin study of 9- and 12-year-old children. Sci. World J..

[20] Katzman D., Spettigue W., Holly A., Couturier J., Dominic A., Pei-Yoong L.F., Maguire B.L., Mawjee K., Parikh S., Steinegger C. (2021). Incidence and Age- and Sex-Specific Differences in the Clinical Presentation of Children and Adolescents With Avoidant Restrictive Food Intake Disorder. JAMA Pediatr..

[21] Scaglioni S., Arrizza C., Vecchi F., Tedeschi S. (2011). Determinants of children’s eating behavior. Am. J. Clin. Nutr..

[22] Mazzeo S.E., Zucker N.L., Gerke C.K., Mitchell K.S., Bulik C.M. (2005). Parenting concerns of women with histories of eating disorders. Int. J. Eat. Disord..

[23] Inoue T., Otani R., Iguchi T., Ishii R., Uchida S., Okada A., Kitayama S., Koyanagi K., Suzuki Y., Suzuki Y. (2021). Prevalence of autism spectrum disorder and autistic traits in children with anorexia nervosa and avoidant/restrictive food intake disorder. BioPsychoSocial Med..

[24] Norris M.L., Obeid N., Santos A., Valois D.D., Isserlin L., Feder S., Spettigue W. (2021). Treatment needs and rates of mental health comorbidity in adolescent patients with ARFID. Front. Psychiatry.

[25] Kambanis P.E., Kuhnle M.C., Wons O.B., Jo J.H., Keshishian A.C., Hauser K., Becker K.R., Franko D.L., Misra M., Micali N. (2020). Prevalence and correlates of psychiatric comorbidities in children and adolescents with full and subthreshold avoidant/restrictive food intake disorder. Int. J. Eat. Disord..

[26] Dinkler L., Yasumitsu-Lovell K., Eitoku M. (2021). Early neurodevelopmental problems and risk for avoidant/restrictive food intake disorder (ARFID) in the general child population: A Japanese birth cohort study. medRxiv.

[27] Birch L.L., Doub A.E. (2014). Learning to eat: Birth to age 2 years. Am. J. Clin. Nutr..

[28] Keren M., Zeanah C. (2019). Eating and Feeding Disorders in Early Childhood. Handbook of Infant Mental Health.

[29] Cummings E., Melissa R.W., Kalsea G., Koss J., Davies P. (2013). Parental Depressive Symptoms and Adolescent Adjustment: Responses to Children’s Distress and Representations of Attachment as Explanatory Mechanisms. Parenting.

[30] Cerniglia L., Marzilli E., Cimino S. (2020). Emotional-Behavioral Functioning, Maternal Psychopathologic Risk and Quality of Mother-Child Feeding Interactions in Children with Avoidant/Restrictive Food Intake Disorder. Int. J. Environ. Res. Public Health.

[31] Jameson M., Fehringer K., Neu M. (2017). Comparison of two tools to assess dyad feeding interaction in infants with gastroesophageal reflux disease. J. Spéc. Pediatr. Nurs..

[32] Green S., Glanz K. (2015). Development of the Perceived Nutrition Environment Measures Survey. Am. J. Prev. Med..

[33] Leonidas C., Souza A., Azevedo L., Ferreira I., Pessa R., Santos M. (2020). Emotional and feeding aspects of avoidant/restrictive food intake disorder (ARFID): A case report. Int. J. Psychiatry.

[34] Norris M.L., Spettigue W.J., Katzman D.K. (2016). Update on eating disorders: Current perspectives on avoidant/restrictive food intake disorder in children and youth. Neuropsychiatr. Dis. Treat..

[35] Thomas J., Lawson E.A., Micali N., Misra M., Deckersbach T., Eddy K. (2017). Avoidant/Restrictive Food Intake Disorder: A Three-Dimensional Model of Neurobiology with Implications for Etiology and Treatment. Curr. Psychiatry Rep..

[36] Reba-Harrelson L., Von Holle A., Hamer R.M., Torgersen L., Reichborn-Kjennerud T., Bulik C.M. (2010). Patterns of maternal feeding and child eating associated with eating disorders in the Norwegian Mother and Child Cohort Study (MoBa). Eat. Behav..

[37] Russell G.F., Treasure J., Eisler I. (1998). Mothers with anorexia who underfeed their children: Their recognition and management. Psychol. Med..

[38] Agras S., Hammer L., McNicholas F. (1999). A prospective study of the influence of eating disordered mothers on their children. Int. J. Eat. Disord..

[39] Micali N., Simonoff E., Stahl D., Treasure J. (2009). Infant feeding and weight in the first year oflife in babies of women with eating disorders. J. Pediatr..

[40] Micali N., Simonoff E., Stahl D., Treasure J. (2011). Maternal eating disorders and infant feeding difficulties: Maternal and child mediators in a longitudinal general population study. J. Child Psychol. Psychiatry.

[41] Birch L.L., Davison K.K. (2001). Family environmental factors influencing the developing behavioral controls of food intake and childhood overweight. Pediatr. Clin. N. Am..

[42] Braden A., Rhee K., Peterson C.B., Rydell S.A., Zucker N., Boutelle K. (2014). Associations between child emotional eating and general parenting style, feeding practices, and parent psychopathology. Appetite.

[43] Cimino S., Cemiglia L., Porreca A., Simonelli A., Ronconi L., Ballarotto G. (2016). Mothers and fathers with binge eating disorder and their 18–36 months old children: A longitudinal study on parent–infant interactions and offspring’s emotional– behavioral profiles. Front. Psychol..

[44] Chatoor I., Ganiban J., Hirsch R., Borman-Spurrell E., Mrazek D.A. (2000). Maternal Characteristics and Toddler Temperament in Infantile Anorexia. J. Am. Acad. Child Adolesc. Psychiatry.

[45] Ziółkowska B., Ocalewski J., Zickgraf H., Brytek-Matera A. (2022). The Polish Version of the Avoidant/Restrictive Food Intake Disorder Questionnaire—Parents Report (ARFID-Q-PR) and the Nine Items Avoidant/Restrictive Food Intake Disorder Screen—Parents Report (NIAS-PR): Maternal Perspective. Nutrients.

[46] Wardle J., Sanderson S., Guthrie C.A., Rapoport L., Plomin R. (2002). Parental feeding style and the inter-generational transmission of obesity risk. Obesity.

[47] Wojciechowska J. (2019). Rodzina Przy Stole. Analiza Zachowań Rodziców i Dzieci.

[48] Lo K., Cheung C., Lee A., Tam W., Keung V. (2015). Assotiations between parental feeding styles and childhood eating habits: A survey of Hong Kong pre-school children. PLoS ONE.

[49] Grilo C.M., Reas D.L., Hopwood C.J., Crosby R.D. (2015). Factor structure and construct validity of the Eating Disorder Examination-Questionnaire in college students: Further support for a modified brief version. Int. J. Eat. Disord..

[50] Kaiser H.F. (1958). The varimax criterion for analytic rotation in factor analysis. Pyrometrical.

[51] Field A. (2009). Discovering Statistics Using IBM SPSS Statistic.

[52] Ledford J.R., Gast D.L. (2006). Feeding problems in children with autism spectrum disorders: A review. Focus. Autism Other Dev. Disabl..

[53] Nygren G., Linnsand P., Hermansson J., Gillberg C. (2021). Disorder in Young Children With Autism Spectrum Disorder in a Multiethnic Population. Front. Pediatr..

[54] Sharp W.G., Berry R.C., McCracken C., Nuhu N.N., Marvel E., Saulnier C.A., Klin A., Jones W., Jaquess D. (2013). Feeding problems and nutrient intake in children with autism spectrum disorders: A meta-analysis and comprehensive review of the literature. J. Autism Dev. Disord..

[55] Boerner K.E., Coelho J.S., Syal F., Bajaj D., Finner N., Dhariwal A.K. (2022). Pediatric Avoidant-Restrictive Food Intake Disorder and gastrointestinal-related Somatic Symptom Disorders: Overlap in clinical presentation. Clin. Child Psychol. Psychiatry.

[56] Burton Murray H., Bailey A., Keshishian A., Eddy K., Thomas J.J., Kuo B. (2019). Prevalence and Characteristics of Avoidant/Restrictive Food Intake Disorder in Adult Neurogastroenterology Patients. Clin. Gastroenterol. Hepatol..

[57] Greer F.R., Sicherer S.H., Burks W., Committee on Nutrition and Section on Allergy and Immunology (2008). Effects of early nutritional interventions on the development of atopic disease in infants and children: The role of maternal dietary restriction, breastfeeding, timing of introduction of complementary foods, and hydrolyzed formulas. Pediatrics.

[58] Eow S.Y., Gan W.Y., Lim P.Y., Awang H., Mohd Shariff Z. (2022). Parental Feeding Practices and Child-Related Factors are Associated with Overweight and Obesity in Children and Adolescents with Autism Spectrum Disorder. J. Autism Dev. Disord..

[59] Diolordi L., del Balzo V., Bernabei P., Vitiello V., Donini L. (2014). Eating habits and dietary patterns in children with autism. Eat. Weight Disord..

[60] Martin C.I., Dovey T.M., Coulthard H., Southall A. (2013). Maternal stress and problem-solving skills in a sample of children with non-organic feeding disorders. Infant Ment. Health J..

[61] Didden R., Seys D., Schouwink D. (1999). Treatment of chronic food refusal in a young developmentally disabled child. Behav. Interv..

[62] Piazza C., Fisher W., Brown K.A., Shore B., Patel M., Katz R., Sevin B., Gulotta C., Blakely-Smith A. (2003). Functional analysis of inappropriate mealtime behaviors. J. Appl. Behav. Anal..

[63] Blissett J., Haycraft E. (2011). Parental eating disorder symptoms and observations of mealtime interactions with children. J. Psychosom. Res..

[64] Martini M., Barona-Martinez M., Micali N. (2020). Eating disorders mothers and their children: A systematic review of the literature. Arch. Women’s Ment. Health.

[65] Hoffman E., Bentley M., Hamer R., Hodges E., Ward D., Bulik M. (2012). A comparison of infant and toddler feeding practices of mothers with and without histories of eating disorders. Matern. Child Nutr..

